# Cost-effectiveness of digital breast tomosynthesis versus digital mammography for breast cancer screening: a systematic literature review

**DOI:** 10.2478/abm-2026-0025

**Published:** 2026-08-31

**Authors:** Supakorn Chansaengpetch, Lisa Stirk, Nigel Armstrong

**Affiliations:** Department of Economics and Related Studies, University of York, York, YO10 5DD, United Kingdom; Department of Preventive and Social Medicine, Faculty of Medicine, Chulalongkorn University, Bangkok 10330, Thailand; Kleijnen Systematic Reviews Ltd, York, YO19 6FD, United Kingdom

**Keywords:** breast neoplasms, cost-effectiveness, digital breast tomosynthesis, early detection of cancer, mammography

## Abstract

**Background:**

Digital breast tomosynthesis (DBT) is being rapidly adopted and has the potential to improve diagnostic performance compared with digital mammography (DM), the current standard for breast cancer screening. However, its cost-effectiveness remains uncertain, and a systematic synthesis of economic evidence is lacking. Therefore, this study aims to review existing cost-effectiveness analyses of DBT versus DM in average-risk women to inform policy decision-making and identify key considerations for future economic evaluations.

**Methods:**

Seven databases (MEDLINE, Embase, KSR Evidence, Cochrane Library, NHSEED, CEA Registry, and RePEc IDEAS) were searched in June 2025. Eligible studies included cost-utility analysis and cost-effectiveness analysis of DBT compared with DM alone in women at average risk of breast cancer. Outcomes included costs and effectiveness, measured in quality-adjusted life years or life years gained. Study characteristics, modeling approaches, and cost-effectiveness results were extracted and narratively synthesized.

**Results:**

A total of 10 studies published between 2015 and 2025 were included, 5 assessing DBT with DM and 5 assessing standalone DBT. Conclusions varied from cost-saving to not cost-effective. Studies including women aged 40–74 years and modeling biennial screening generally found DBT to be cost-effective. Evidence for standalone DBT was limited but suggested potential cost savings in some settings.

**Conclusions:**

DBT may be a cost-effective intervention compared with DM for breast cancer screening. However, further robust economic evaluations, particularly of standalone DBT, are warranted to inform policy decision-making across diverse healthcare settings, particularly within Asian settings.

Breast cancer remains a leading cause of cancer incidence and cancer-related mortality among women worldwide [1]. In 2022, it was the most frequently diagnosed cancer in women in 183 of 185 countries and the second most frequently diagnosed in the remaining countries, with an estimated 2.3 million new cases and 670,000 deaths. This accounted for approximately 25% of all new cancer diagnoses and 15.5% of global cancer-related deaths [2]. Projections indicate that, by 2050, annual incidence will increase by 38% and mortality by 68% [2]. Furthermore, between 2020 and 2030, both mortality rates and disability-adjusted life year rates are expected to rise, particularly among women aged 40–49 years and 45–49 years, respectively [3]. In Asia, breast cancer incidence has also continued to increase steadily [4]. Importantly, early detection through population-based screening programs has contributed to reductions in both mortality and the incidence of advanced disease in multiple countries, highlighting its critical role in breast cancer control strategies [5, 6].

Cancer screening is designed to detect malignancies at an early, more treatable stage [7]. In breast cancer, digital mammography (DM), also known as two-dimensional mammography, remains the most widely recommended screening modality, including across several Asian countries. DM uses low-dose X-ray imaging to detect lesions before they are clinically detectable [4, 5, 8]. Compared with other cancers, localized breast cancer has an excellent prognosis, with 5-year relative survival rates reaching up to 99% [9]. A meta-analysis of randomized controlled trials (RCTs) has demonstrated that mammographic screening reduces breast cancer mortality by approximately 19% [8].

Despite these benefits, screening with DM is associated with important limitations, including overdiagnosis and false-positive findings, which may lead to unnecessary biopsies and psychological distress [7, 10]. In clinical practice, individuals with incomplete assessments or suspicious to highly suggestive findings typically require additional diagnostic evaluation [11]. UK data indicate that approximately 3.4% of screened women undergoing DM are recalled for false-positive findings requiring biopsy [7]. Moreover, the sensitivity of DM is reduced in women under 50 years of age and in those with dense breast tissue, which is present in approximately 50% of women in Western populations and up to 70% of women in Asian populations [12, 13]. A key limitation of DM is the superimposition of dense fibroglandular breast tissue, which can obscure lesions and consequently reduce both diagnostic performance and cancer detection rates [12].

Digital breast tomosynthesis (DBT) is an emerging mammography technology that produces pseudo-3-dimensional breast images [12, 14]. Over the past decade, it has been rapidly adopted into routine clinical practice [14]. DBT acquires multiple low-dose X-ray projections as the X-ray tube moves along an arc over a defined angular range, which are then reconstructed into a composite 3-dimensional representation. Its overall mean glandular dose is comparable to that of conventional DM [12]. DBT can be performed either in combination with DM or as a standalone modality using synthesized two-dimensional (s2D) images, with the latter associated with a lower radiation dose [14].

By reducing the impact of overlapping dense fibroglandular tissue, DBT addresses a key limitation of DM and has the potential to improve diagnostic performance in breast cancer screening [12]. Meta-analyses have demonstrated that DBT increases cancer detection rates by 1.6–3.2 per 1,000 women screened and reduces recall rates for further assessment by approximately 2.2% compared with DM [15, 16]. Because false-positive findings constitute a substantial proportion of screening recalls [15], reductions in recall rates are likely to reflect meaningful decreases in false-positive results. A prospective US cohort study reported that the 10-year cumulative probability of a false-positive recall was significantly lower with DBT than with DM, with an absolute difference of up to 6.7% [17]. Another systematic review also found lower false-positive rates for DBT combined with DM (DBT-DM) compared with DM alone [18]. In addition, a prospective cohort study in Sweden demonstrated superior sensitivity for DBT relative to DM (81.1% versus 60.4%) [19]. Improved sensitivity has also been observed in women with dense breasts, with reported increases from 84% to 90% for DBT compared with 69%–86% for DM in diagnostic settings [20].

DM remains cost-effective across diverse healthcare settings worldwide [21, 22]. In Asia, population-based mammography screening programs have been implemented in countries such as Japan, South Korea, Singapore, and Taiwan [4]. Despite the clinical advantages of DBT, its economic value in population-based screening programs remains uncertain. Although DBT generally entails higher upfront costs than DM, these costs may be partially or fully offset by reductions in downstream expenditures, including fewer false-positive recalls, fewer unnecessary diagnostic procedures, and improved early cancer detection [13, 23]. Cost-effectiveness analysis is therefore essential for informing health policy, as it evaluates the balance between incremental costs and health outcomes across competing technologies. In this context, it helps determine whether the health gains associated with DBT justify its additional costs relative to the current standard of care, DM, while accounting for health opportunity costs.

To date, systematic reviews synthesizing global evidence on the cost-effectiveness of DBT screening in women at average risk remain limited. A systematic review of cost-effectiveness studies on breast cancer prevention and treatment published between 2015 and 2019 identified only two studies evaluating DBT screening, both conducted in the United States [22, 24, 25]. These studies suggested that DBT could be cost-effective at commonly used willingness-to-pay (WTP) thresholds. Another systematic review focusing on economic evaluations of mammography screening in low- and middle-income countries (LMICs) identified 21 studies published between 2006 and 2021, none of which assessed DBT [26]. More recent evidence, however, presents mixed findings, with some studies suggesting that DBT may be cost-effective or even cost-saving compared with DM [13, 23, 27].

Given the rapid diffusion of DBT and the substantial global economic burden of breast cancer [3, 12], there is an urgent need to evaluate its cost-effectiveness in population-based screening settings and to examine how methodological choices and study characteristics influence reported outcomes. A systematic review of cost-effectiveness analyses (CEA) comparing DBT and DM would therefore address a critical policy gap across diverse healthcare systems and guide future economic evaluations, clinical practice, and health policy decision-making.

This systematic review aims to identify and synthesize CEA comparing DBT (with or without DM) versus DM alone for breast cancer screening in women at average risk. It further aims to examine how study characteristics and modeling approaches influence cost-effectiveness results.

## Methods

This systematic review of CEA was conducted in accordance with the methodological guidance provided in the Cochrane Handbook for Systematic Reviews of Interventions [28] and the Centre for Reviews and Dissemination (CRD) guidance for undertaking reviews in health care [29]. Although the review was not formally registered and a protocol was not prepared, the eligibility criteria and search strategy were established a priori, before the initiation of the search, and no post hoc amendments were made.

### Eligibility criteria

The eligibility criteria are summarized in **Table 1**. The study population comprised asymptomatic individuals assigned female at birth, with no restrictions on age. Exclusion criteria were informed by eligibility frameworks used in previous evidence syntheses on breast cancer screening conducted by international review bodies [30, 31]. DBT was defined as the intervention of interest, regardless of whether it was used in combination with DM or with s2D mammography. The comparator was DM alone, which remains the current standard primary screening modality and is widely recommended for women at average risk [5]. Eligible studies were required to report both cost and effectiveness outcomes and/or the incremental cost-effectiveness ratio (ICER) comparing the two screening strategies. Health outcomes of interest included quality-adjusted life years (QALYs) and/or life years gained (LYG). Only full economic evaluations were included, specifically cost-utility analyses (CUA) and CEA. Gray literature was excluded to enhance consistency and transparency in reporting across included studies.

**Table 1.** Eligibility criteria

**PICOS**	**Inclusion criteria**	**Exclusion criteria**
Population	Asymptomatic female	- History of breast cancer or high-risk lesions (i.e., DCIS, LCIS, ADH, ALH) - Presence of significant genetic predispositions or syndromes associated with high risk for breast cancer (e.g., BRCA1/2 gene mutations, Li-Fraumeni syndrome) - History of therapeutic chest radiation (20 Gy or more) before age 30 years
Intervention	DBT with/without DM	- Imaging is specifically used for diagnostic or surveillance purposes
Comparator	DM alone	- DM combined with any other supplemental screening modality
Outcomes	- Costs - Effectiveness outcomes (QALYs or LYs) - Incremental costs - Incremental effectiveness (QALYs or LYs) - ICER	- NA
Study designs	Full economic evaluation, including CUA and CEA	- CBA - Partial economic evaluation
Limit	Peer-reviewed articles	- Gray literature - Editorials, reviews, and commentaries - No full-text available

ADH, atypical ductal hyperplasia; ALH, atypical lobular hyperplasia; CBA, cost-benefit analysis; CEA, cost-effectiveness analysis; CUA, cost-utility analysis; DBT, digital breast tomosynthesis; DCIS, ductal carcinoma in situ; DM, digital mammography; Gy, gray (unit); ICER, incremental cost-effectiveness ratio; LCIS, lobular carcinoma in situ; LYs, life years; NA, not applicable; QALYs, quality-adjusted life years.

### Search strategy

A comprehensive literature search was conducted in MEDLINE (Ovid), Embase (Ovid), KSR Evidence (EB Health), and the Cochrane Library (Wiley), focusing on the intervention of interest (DBT) (see **Appendix 1**). For example, in Embase, search terms included controlled vocabulary and free-text keywords such as *digital breast tomosynthesis/*, “3-D mammogra$,” “3D mammogra$,” “three-dimensional mammogra$,” and “3-dimensional mammogra$,” as well as proximity operators such as *tomosynthesis adj6 (breast$ or mammar*$). The search strategy was developed in consultation with an experienced information specialist (LS) to ensure both accuracy and comprehensiveness. Additional terms related to specific DBT systems and manufacturers were also included, such as “3Dimensions,” “Selenia Dimensions,” “Senographe Pristina,” “VolumeRAD,” “Wide-Angle Tomosynthesis,” “Helianthus,” “ASPIRE Cristalle,” “Amulet Innovality,” “Planmed Clarity,” and “Giotto Class.” Methodological filters for economic evaluations were applied using validated search filters derived from the National Health Service Economic Evaluation Database (NHS EED) for both Embase and MEDLINE, obtained from the CRD website (https://www.crd.york.ac.uk/crdweb/searchstrategies.asp). No restrictions were placed on publication date or language.

To identify additional gray literature, supplementary searches were conducted in the NHSEED database (https://www.crd.york.ac.uk/CRDWeb/), the CEA Registry (https://cear.tuftsmedicalcenter.org), and the RePEc IDEAS database (https://ideas.repec.org) using keywords related to tomosynthesis and 3-dimensional mammography. All databases were searched on June 19, 2025 (**Table 2**). In addition, the reference lists of all eligible studies were manually screened to identify any further relevant publications.

**Table 2.** Databases searched

**Databases**	**Date parameters**
MEDLINE via Ovid	1946–June 18, 2025
Embase via Ovid	1974–June 18, 2025
EB Health –KSR Evidence via Ovid	2015–2025 week 24
Cochrane Library via Wiley	Inception to present
NHS EED via https://www.crd.york.ac.uk/CRDWeb/	Inception to March 2015
CEA registry via https://cear.tuftsmedicalcenter.org	Inception to present
RePEc IDEAS via https://ideas.repec.org	Inception to present

CEA, cost-effectiveness analyses; CRD, Centre for Reviews and Dissemination; NHS EED, National Health Service Economic Evaluation Database.

### Screening and study selection

One author (SC) independently screened the titles and abstracts of all identified records to determine their potential eligibility. Following this initial stage, full texts of all potentially relevant studies were retrieved and assessed in detail against the predefined eligibility criteria. It is important to note that, due to dissertation requirements, screening and full-text review were conducted by a single reviewer, which deviates from standard methodological best practice, which typically recommends independent dual screening to minimize selection bias. To mitigate the risk of subjective bias and enhance the rigor of the selection process, several safeguards were implemented. The project supervisor (NA), an experienced health economist, reviewed and approved the eligibility criteria prior to the commencement of screening. In addition, screening progress and challenging cases were discussed during regular supervisory meetings, and NA provided adjudication and guidance on all borderline or ambiguous inclusion decisions. Furthermore, records with unclear relevance at the title and abstract stage were deliberately retained for full-text review rather than being excluded, thereby reducing the likelihood of premature or erroneous exclusion of eligible studies. The application of clearly defined, objective inclusion criteria—restricted to full economic evaluations (CUA and CEA) reporting QALYs or life years (LYs)—further minimized the potential for subjective interpretation during study selection.

### Data extraction

A standardized data extraction form was developed a priori to ensure methodological consistency, enhance transparency, and improve the validity and reliability of the extracted evidence [29]. The form was grounded in the review question and guided by the British Medical Journal (BMJ) checklist for economic evaluations [32]. Data were independently extracted by SC into Microsoft Excel. Each study was assigned a unique record number and referenced by the first author’s surname and year of publication. Extracted data included general study characteristics, population of analysis, and details of the intervention, model structure, model parameterization, base-case economic evaluation results, and sensitivity analyses.

### Assessment of reporting quality

The reporting quality of the included studies was assessed using the BMJ checklist for economic evaluations [32], which emphasizes transparency, completeness, and appropriateness in the reporting of health economic evaluations. The checklist comprises 35 items (see **Appendix 2**). Each item was rated as yes, no, “not clear,” or “not applicable.” The proportion of items rated as yes was used as an indicator of reporting quality for each included study in this review. It is important to note that this checklist is not designed to assess methodological quality or to generate an overall quality score.

### Data synthesis

A narrative synthesis of the included studies was undertaken and supported by a tabular presentation of key findings. Data were primarily synthesized according to general study characteristics, intervention types, and methodological approaches. For economic evaluation outcomes, studies were categorized by intervention type (i.e., DBT with or without DM). An intervention was considered cost-effective if the ICER fell below the WTP threshold specified in each study, whether expressed in QALYs or LYs. To facilitate comparability across studies, ICERs were further standardized to 2023 international dollars (I$) by inflating costs to 2023 in local currency units using the gross domestic product implicit price deflator, followed by conversion using the 2023 purchasing power parity conversion factor. For sensitivity analyses, the most influential parameter affecting the ICER in each study was extracted to represent the primary driver of cost-effectiveness, along with the corresponding range or alternative assumption reported. Given substantial heterogeneity in healthcare systems, population characteristics, modeling approaches, and outcome measures, a quantitative meta-analysis was not undertaken.

## Results

### Study selection

A total of 829 records were initially identified, of which 206 duplicates were removed. After title and abstract screening, 20 records were retrieved for full-text assessment for eligibility. Of these, 10 records were excluded for the following reasons: not reporting QALYs or LYG (n = 1) [33]; not being a CEA or CUA (n = 4) [34,35,36,37]; conference abstract (n = 4) [38,39,40,41]; and health technology assessment (HTA) reports (n = 1) [42]. Ultimately, 10 studies met the eligibility criteria and were included in the review. All included studies were published in English. **Figure 1** presents the PRISMA flow diagram [43] detailing the study identification and selection process.

**Figure 1.**
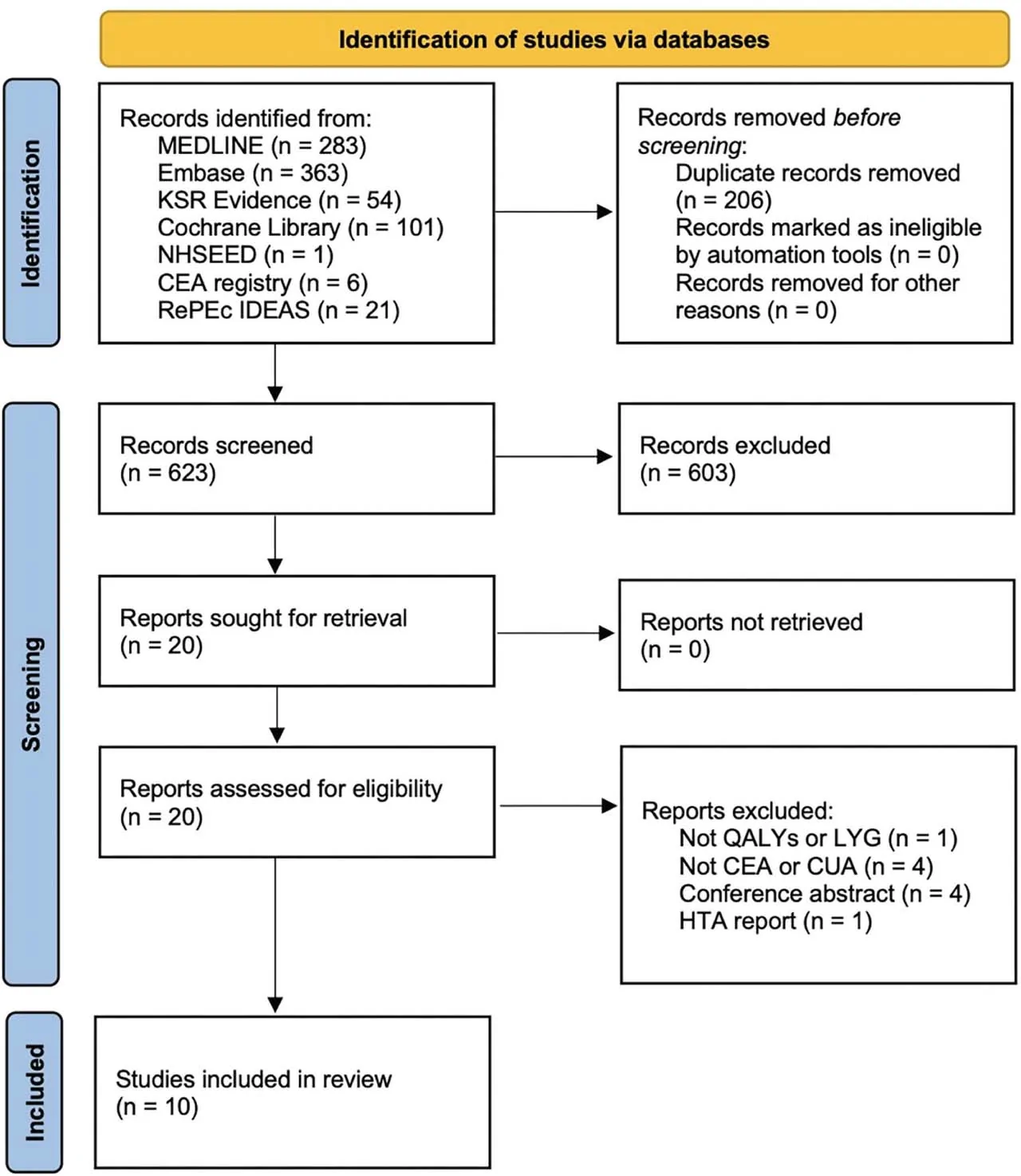
PRISMA flow diagram for study selection. CEA, cost-effectiveness analyses; CUA, cost-utility analyses; HTA, health technology assessment; LYG, life years gained; NHSEED, National Health Service Economic Evaluation Database.

### Characteristics of included studies

The characteristics of the included studies are summarized in **Table 3**. Of the 10 studies, 8 were published between 2020 and 2025 [13, 23, 44,45,46,47,48,49], while the remaining 2 were published between 2015 and 2016 [24, 25]. In terms of geographic distribution, most studies were conducted in Europe (the Netherlands [n = 2], Sweden [n = 1], and Norway [n = 1]) [45, 46, 48, 49] and North America (the United States [n = 3] and Canada [n = 1]) [24, 25, 44, 47]. The remaining studies were conducted in Taiwan [13] and Brazil [23]. All included studies were model-based analyses simulating organized population-based breast cancer screening programs.

**Table 3.** Characteristics of included studies

**Publication**	**Country**	**Study population**	**Intervention**	**Screening interval**	**Primary economic evaluation outcome**	**Industry funding**
Chung et al. 2024 [13]	Taiwan	Women aged 40–69 years	DBT-DM	Biennial	ICER	No
Couto et al. 2024 [23]	Brazil	Women aged 40–69 years with BI-RADS B or C	DBT with s2D	Biennial	ICER	Siemens Healthineers
Cressman et al. 2021 [47]	Canada	Women aged 40–74 years	DBT-DM	Biennial	ICER	No
Fridhammar et al. 2025 [49]	Sweden	Women aged 40–74 years	DBT	1.5–2 years	Relative reduction in breast cancer mortality for DBT to be cost effective	No
Kalra et al. 2016 [25]	USA	Women aged 40–79 years	DBT-DM	Annual	ICER	No
Lee et al. 2015 [24]	USA	Women aged 50–74 years with dense breasts	DBT-DM	Biennial	ICER	No
Lowry et al. 2020 [44]	USA	Women aged 40–79 years	DBT-DM	1–2 years	ICER	No
Moger et al. 2022 [48]	Norway	Women aged 50–69 years	DBT with s2D	Biennial	ICER	No
Sankatsing et al. 2020 [45]	Netherlands	Women aged 50–74 years	DBT	Biennial	ICER	No
Wang et al. 2020 [46]	Netherlands	Women aged 50–75 years	DBT	Biennial	Sensitivity at which DBT would be cost effective	No

DBT, digital breast tomosynthesis; DBT-DM, DBT combined with DM; DM, digital mammography; ICER, incremental cost-effectiveness ratio; s2D, synthesized two-dimensional mammography.

### Population of analysis

Most studies included asymptomatic women initiating screening at age 40 (n = 6); however, age ranges varied considerably across studies. Two studies targeted women aged 40–69 years [13, 23], two focused on women aged 40–74 years [47, 49], and another two included women aged 40–79 years [25, 44]. The remaining 4 studies initiated screening at age 50, including one study focusing on women aged 50–69 years [48] and 3 studies including women aged 50–74 years or 75 years [24, 45, 46]. Although most studies evaluated the general population of asymptomatic women, two studies specifically focused on populations stratified by breast density. One study included women with scattered areas of fibroglandular density and heterogeneously dense breasts [23], while the other was restricted to women with dense breast tissue, specifically heterogeneously and extremely dense breasts [24]. This subgroup focus reflects an increasing recognition of breast density as a key modifier of screening performance and downstream cost-effectiveness outcomes.

### Intervention description

Half of the included studies evaluated DBT-DM [13, 24, 25, 44, 47], while the remaining half assessed DBT as a standalone screening modality [23, 45, 46, 48, 49]. Biennial screening was the most frequently reported interval, applied in 7 studies [13, 23, 24, 45,46,47,48]. One study employed annual screening [25], whereas two studies reported variable screening intervals, ranging from 1.5 years to 2 years [49] and 1–2 years [44], respectively. Importantly, all included studies used DM alone as the comparator, with screening intervals generally aligned to those used in the intervention arm within each respective model.

### Primary economic outcome

Eight studies reported the ICER as their primary economic outcome. Of these, 4 studies reported ICERs exclusively in terms of QALYs [13, 25, 44, 47], two reported ICERs solely in terms of LYs [45, 48], and two presented ICERs using both QALYs and LYs [23, 24]. In contrast, the remaining two studies adopted threshold-based approaches rather than conventional ICER reporting. Specifically, the Swedish study estimated the minimum relative reduction in breast cancer mortality required for DBT to be considered cost-effective under predefined WTP thresholds [49], while the Dutch study identified the minimum diagnostic sensitivity at which DBT would achieve cost-effectiveness [46].

### Model characteristics and parameterization

An overview of the model characteristics and parameterization for each included study is presented in **Table 4.** The economic evaluations comprised 7 CUAs [13, 23,24,25, 44, 47, 49] and 3 CEAs [45, 46, 48]. Most studies adopted a payer perspective [23,24,25, 44, 47, 49], whereas the Taiwanese study was the only one to adopt a societal perspective [13]. Nine studies employed long-term analytic horizons, ranging from 30 years to 40 years [13, 23, 47] or extending over a lifetime horizon [24, 25, 44,45,46, 49]. In contrast, one study adopted a shorter 10-year time horizon [48]. Regarding discounting practices, all included studies applied equal discount rates for both costs and health outcomes in their base-case analyses, with rates ranging from 2% [13] to 5% [23].

**Table 4.** Model characteristics

**Publication**	**Evaluation type**	**Model type**	**Perspective**	**Time horizon (years)**	**Discount rate (%)**	**Cost estimation**	**Currency, cost year**
Chung et al. 2024 [13]	CUA	State transition cohort model	Societal	30	2	Direct and indirect	$, 2021
Couto et al. 2024 [23]	CUA	Decision tree with state transition model	Payer	30	5	Direct	€, 2023
Cressman et al. 2021 [47]	CUA	State transition cohort model	Payer	40	3	Direct	C$, 2019
Fridhammar et al. 2025 [49]	CUA	Unclear	Payer	Lifetime	3	Direct	€, 2022
Kalra et al. 2016 [25]	CUA	State transition cohort model	Payer	Lifetime	3	Direct	$, 2015
Lee et al. 2015 [24]	CUA	Discrete event simulation	Payer	Lifetime	3	Direct	$, 2013
Lowry et al. 2020 [44]	CUA	Discrete event simulation and analytical approach	Payer	Lifetime	3	Direct	$, 2018
Moger et al. 2022 [48]	CEA	Decision tree model	NR	10	4	Direct	€, 2018
Sankatsing et al. 2020 [45]	CEA	Microsimulation	NR	Lifetime	3.5	Direct	€, 2020
Wang et al. 2020 [46]	CEA	Microsimulation	NR	Lifetime	3	Direct	€, NR

$, US dollar; €, Euro; C$, Canadian dollar; CEA, cost-effectiveness analysis; CUA, cost-utility analysis; NR, not reported.

### Modeling approaches

Several modeling approaches were identified across the included studies. Three studies employed state-transition cohort models [13, 25, 47], while one study used a decision tree model [48]. Another study adopted a hybrid framework combining a decision tree with a state-transition model [23]. Two Dutch studies applied validated microsimulation models, namely the Microsimulation SCreening ANalysis model [45] and the simulation model on radiation risk and breast cancer screening [46]. Similarly, two US studies used discrete-event simulation models based on the validated cancer intervention and surveillance modeling network (CISNET) framework [24, 44]. One study did not explicitly specify the underlying model type; however, the authors reported using a health economic model developed in Microsoft Excel to inform the national breast cancer screening guidelines in Sweden [49].

### Test performance of mammographic screenings

**Table 5** summarizes the test performance characteristics and data sources for both intervention and comparator strategies across the included studies, including measures such as sensitivity, specificity, and recall rates. Six studies reported recall rates [13, 23, 25, 47,48,49], whereas the remaining 4 studies reported sensitivity and/or specificity measures [24, 44,45,46].

**Table 5.** Test performance of mammographic screenings reported

**Publication**	**Sensitivity (%)**		**Specificity (%)**		**Recall rate (%)**		**Source(s)**
		
	**Intervention**	**Comparator**	**Intervention**	**Comparator**	**Intervention**	**Comparator**	
Chung et al. 2024 [13]	–	–	–	–	9.0	11.8	Retrospective cohort study [52]
Couto et al. 2024 [23]	–	–	–	–	3.5	4.2	Meta-analysis [23]
Cressman et al. 2021 [47]	–	–	–	–	Absolute reduction of 2.2		Meta-analysis [15]
Fridhammar et al. 2025 [49]	–	–	–	–	3.6	2.5	Prospective cohort study [19]
Kalra et al. 2016 [25]	–	–	–	–	9.1	10.7	Retrospective cohort study [53]
Lee et al. 2015 [24]	80	77	92	88	–	–	Prospective cohort study [54]
Lowry et al. 2020 [44]	84.3–96.8	88.2–95.8	76.6–91.1	65.5–84.3	–	–	Prospective cohort study [55, 56]
Moger et al. 2022 [48]	–	–	–	–	4.4	4.3	RCT [50]
Sankatsing et al. 2020 [45]	18 increase		–	–	–	–	Expert estimation and prospective cohort studies [57, 58]
Wang et al. 2020 [46]	65–100	65–87	96.5	96.5	–	–	DBT performance: assumption; DM sensitivity: retrospective cohort study [59]; DM specificity: RCT [60]

DBT, digital breast tomosynthesis; DM, digital mammography; RCT, randomized controlled trial.

Overall, the intervention strategies, whether DBT alone or DBT-DM, generally demonstrated lower recall rates compared with DM alone. For example, Chung et al. [13] reported recall rates of 9.0% for DBT-DM and 11.8% for DM alone. Similarly, Couto et al. [23] reported recall rates of 3.5% versus 4.2%, while Kalra et al. [25] reported rates of 9.1% and 10.7% for DBT-DM and DM, respectively. Cressman et al. [47] further reported an absolute recall rate reduction of 2.2% associated with DBT-DM. In contrast, Moger et al. [48], using data derived from the To-Be trial [50], reported comparable overall recall rates between DBT and DM (4.4% versus 4.3%). Nevertheless, DBT demonstrated a lower recall rate following a prior DM screen (2.94%) compared with repeat DM screening in the To-Be1 study (3.99%) [48, 51]. These findings suggest that the magnitude of recall reduction associated with DBT may vary according to screening history, population characteristics, and implementation context.

### Base-case economic evaluation results

Among the studies assessing DBT-DM (n = 5) **(Table 6)**, 4 concluded that the intervention was cost-effective [13, 24, 25, 47], with reported ICERs ranging from $5,972 [13] to $53,893 [24] per QALY gained. The corresponding incremental net monetary benefits (NMBs) ranged from $351 [24] to $3,188 [25]. Notably, subgroup analysis in one US study demonstrated that women aged 40–49 years achieved approximately threefold higher NMBs compared with women aged 50–59 years [25], suggesting greater economic value of DBT-DM among younger screening populations. In contrast to the other US studies [24, 25], Lowry et al. [44] reported that DBT-DM was not cost-effective in any of the evaluated CISNET models. Reported ICERs ranged from $195,026 to $270,135 per QALY gained, substantially exceeding the specified WTP thresholds of $50,000 to $150,000 per QALY gained [44].

**Table 6.** Base-case cost-effectiveness results for DBT-DM

**Publication**	**Intervention**		**Comparator**		**Incremental cost**	**Incremental QALYs**	**ICER (QALYs)**	**ICER threshold (QALYs)**	**ΔNMB**	**ICER (2023)**	**Author’s conclusions on cost-effectiveness**
	
	**Mean QALYs**	**Mean total cost**	**Mean QALYs**	**Mean total cost**							
Chung et al. 2024 [13]	22.994	$7,861.1	22.954	$7,621.7	$239.4	0.04	$5,972	$33,004	$1,084	I$6,624	Cost effective
Cressman et al. 2021 [47]	NR	NR	NR	NR	C$470	0.027	C$17,149	C$100,000	C$2,230	I$17,930	Cost effective
Kalra et al. 2016 [25]	15.5	$15,312	15.46	$14,500	$812	0.04	$20,300	$100,000	$3,188	I$25,532	Cost effective
Lee et al. 2015 [24]	16.814	$4,440	16.807	$4,091	$349	0.007	$53,893	$100,000	$351	I$70,698	Cost effective
Lowry et al. 2020^†^ [44]	14,914–15,757	$4.6–5.0	14,912–15,756	$4.2–4.6	$0.40–0.45	1.7–2.2	$195,026–270,135	$50,000–150,000	–	I$233,266–323,103	Not cost effective

† Outcomes reported per 1,000 simulated women; costs presented in million.

$, US dollar; ΔNMB, incremental net monetary benefit; C$, Canadian dollar; I$, international dollars; ICER, incremental cost-effectiveness ratio; NR, not reported; QALYs, quality-adjusted life years.

Among studies investigating standalone DBT (n = 5) **(Table 7)** [23, 45, 46, 48, 49], two studies concluded that DBT was a dominant strategy, indicating improved health outcomes at lower overall costs compared with DM alone [23, 48]. A Dutch study reported inconclusive findings, with an ICER of €27,023 per LYG, which fell within the evaluated WTP threshold range of €20,000–35,000 per LYG. Consequently, the corresponding NMB values ranged from −€35,755 to €40,650, reflecting substantial uncertainty in cost-effectiveness estimates under varying threshold assumptions [45].

**Table 7.** Base-case cost-effectiveness results for DBT

**Publication**	**Intervention**		**Comparator**		**Incremental cost**	**Incremental QALYs**	**ICER (QALYs)**	**ICER threshold (QALYs)**	**ΔNMB**	**Author’s conclusions on cost-effectiveness**
	
	**Mean QALYs**	**Mean total cost**	**Mean QALYs**	**Mean total cost**						

Couto et al. 2024 [23]	18.919	€2,094.5	13.720	€3,048.6	–€954.02	5.199	Dominates	€7,200	€38,386	Cost effective (dominant)
Fridhammar et al. 2025^†^ [49]	–613.9 to –581.1	€12.81–13.13	–630.0	€12.32	€0.49–0.80	16.1–48.9	–	€10,000–50,000	–	Cost effective when the relative breast cancer mortality reduction is 3.6%–10.1%

**Publication**	**Intervention**		**Comparator**		**Incremental cost**	**Incremental LYs**	**ICER (LYs)**	**ICER threshold (LYs)**	**ΔNMB**	**Author’s conclusions on cost-effectiveness**
	
	**Mean LYs**	**Mean total cost**	**Mean LYs**	**Mean total cost**						

Moger et al. 2022^‡^ [48]	NR	NR	NR	NR	–€50,000	34.8	Dominates	€ 22,000	€815,600	Cost effective (dominant)
Sankatsing et al. 2020^§^ [45]	73	€443,385	67	€305,829	€137,555	5.09	€27,023 (I$43,311)	€20,000–35,000	–€35,755–40,650	Inconclusive
Wang et al. 2020 [46]	NR	NR	NR	NR	NR	–76–120 (per 10,000)	–	€20,000	–	Cost-effective when sensitivity is at least 95%

† Outcomes presented for a hypothetical cohort of 100,000 women; costs presented in million.

‡ Outcomes presented per 100,000.

§ Outcomes presented per 1,000.

€, Euro; ΔNMB, incremental net monetary benefit; DBT, digital breast tomosynthesis; I$, international dollars; ICER, incremental cost-effectiveness ratio; LYs, life years; NR, not reported; QALYs, quality-adjusted life years.

### Sensitivity analyses and key drivers of cost-effectiveness

**Table 8** summarizes the findings from the sensitivity analyses conducted across the included studies. Six studies performed probabilistic sensitivity analyses (PSAs), reporting probabilities of cost-effectiveness at predefined WTP thresholds ranging from 36% to 100% [13, 23, 25, 45, 47, 48]. Four of these studies reported probabilities of 80% or greater [13, 23, 47, 48], indicating relatively robust findings under parameter uncertainty. By comparison, one US study reported a probability of cost-effectiveness of 63.2% [25], while a Dutch study reported probabilities ranging from 36% at a WTP threshold of €20,000 per LYG to 66% at €35,000 per LYG [45].

**Table 8.** Sensitivity analyses and primary drivers of cost-effectiveness

**Publication**	**Analysis type**	**Probability that DBT is cost-effective (%)**	**Key parameters influencing ICER**		**Structural uncertainty**

			**Parameter**	**Range varied or alternative assumption**	
Chung et al. 2024 [13]	Deterministic and probabilistic	98.5	Recall rate of DBT-DM	[8.1%, 9.9%] (prior literature)	Shorter time horizon (10 years)
Couto et al. 2024 [23]	Deterministic and probabilistic	100	Cancer detection rate of DM	Unclear (±25% variation or 95% CI)	NR
Cressman et al. 2021 [47]	Deterministic and probabilistic	95	Recall rate reduction	[1%, 7.7%] (unclear assumption)	NR
Kalra et al. 2016 [25]	Deterministic and probabilistic	63.2	Unclear	–	NR
Lee et al. 2015 [24]	Deterministic	–	Adjunct DBT cost	[$0, $139] (no additional cost and cost doubled)	NR
Lowry et al. 2020 [44]	Deterministic	–	Adjunct DBT cost	$26 and $36 from base price of $56	NR
Moger et al. 2022 [48]	Deterministic and probabilistic	80	Unclear	–	Extended time horizon (20 years)
Sankatsing et al. 2020 [45]	Deterministic and probabilistic	36–66%	DBT cost	[€72, €125] (expert estimation)	NR

$, US dollar; €, Euro; 95% CI, 95% confidence interval; DBT, digital breast tomosynthesis; DBT-DM: DBT combined with DM; DM, digital mammography; ICER, incremental cost-effectiveness ratio; LYs, life years; NR, not reported; QALYs, quality-adjusted life years.

Deterministic sensitivity analyses (DSAs) showed that the most influential parameters affecting ICER estimates were primarily associated with reductions in recall rates (n = 2) [13, 47] and DBT-related costs (n = 3) [24, 44, 45]. Chung et al. [13] identified the recall rate associated with DBT-DM as the principal driver of cost-effectiveness, while Cressman et al. [47] similarly found recall rate reduction to be the most sensitive parameter within their model. In addition, adjunct DBT costs emerged as major drivers of cost-effectiveness in two US studies [24, 44]. A Dutch study also identified DBT acquisition and implementation costs as highly influential parameters affecting ICER outcomes [45]. Notably, differences in DBT sensitivity were generally not identified as the primary determinant of cost-effectiveness across studies.

The remaining two European studies primarily employed threshold analyses rather than conventional sensitivity analyses [46, 49]. A Swedish study found that DBT would become cost-effective when the relative reduction in breast cancer mortality reached 10.1% at a WTP threshold of €10,000 per QALY gained and 3.6% at a threshold of €50,000 per QALY gained [49]. These thresholds correspond to what Swedish guidelines classify as a moderate cost per QALY gained [49]. Similarly, a Dutch study determined that DBT would be considered cost-effective only if its sensitivity reached at least 95%, assuming a WTP threshold of €20,000 per LYG and a DBT cost of €96 [46]. The authors additionally evaluated an alternative screening strategy in which DBT was offered to women with dense breasts, while women with non-dense breasts continued to receive DM screening. Under this scenario, DBT became cost-effective when sensitivity reached at least 86% [46].

### Study reporting quality assessment

Based on the 35-item BMJ checklist for economic evaluations [32], none of the included studies fulfilled all reporting criteria. The proportion of items rated as yes ranged from 78.1% [13] to 90.3% [23]. All included studies reported conducting sensitivity analyses, either through DSA or PSA. However, 6 studies did not clearly justify the parameter ranges applied within their sensitivity analyses [13, 23, 44, 45, 48, 49]. **Table 9** presents the detailed assessment of reporting quality for all included studies.

**Table 9.** Reporting quality assessment

**Study/Item No.**	1	2	3	4	5	6	7	8	9	10	11	12	13	14	15	16	17	18	19	20	21	22	23	24	25	26	27	28	29	30	31	32	33	34	35	% yes
Chung et al. 2024 [13]	Y	Y	Y	Y	Y	Y	Y	Y	UC	NA	Y	UC	N	Y	Y	N	Y	Y	Y	Y	Y	Y	Y	UC	NA	NA	Y	Y	UC	Y	Y	Y	Y	UC	Y	78.1
Couto etal. 2024 [23]	Y	Y	Y	Y	Y	Y	Y	Y	NA	Y	Y	Y	N	NA	NA	Y	Y	Y	UC	Y	Y	Y	Y	Y	NA	Y	Y	Y	UC	Y	Y	Y	Y	Y	Y	90.3
Cressman etal. 2021 [47]	Y	Y	Y	Y	Y	Y	Y	Y	Y	NA	Y	N	N	NA	NA	N	Y	Y	Y	Y	Y	Y	Y	Y	NA	Y	Y	Y	Y	Y	Y	N	Y	Y	Y	87.1
Fridhammar et al. 2025[49]	Y	Y	Y	Y	Y	Y	Y	Y	Y	NA	Y	UC	Y	NA	NA	N	Y	Y	Y	UC	Y	Y	Y	Y	NA	NA	Y	Y	UC	Y	Y	Y	N	Y	Y	83.3
Kalra et al. 2016 [25]	Y	Y	Y	Y	Y	Y	Y	Y	UC	NA	Y	N	N	NA	NA	N	Y	Y	Y	Y	Y	Y	Y	Y	NA	NA	Y	Y	Y	Y	Y	Y	Y	Y	Y	86.7
Lee et al. 2015 [24]	Y	UC	Y	Y	Y	Y	Y	Y	UC	NA	Y	Y	N	NA	NA	N	Y	Y	Y	Y	Y	Y	Y	Y	NA	NA	Y	Y	Y	Y	Y	Y	Y	Y	Y	86.7
Lowry et al. 2020 [44]	Y	Y	Y	Y	Y	Y	Y	Y	Y	NA	Y	N	N	NA	NA	N	Y	Y	UC	Y	Y	Y	Y	Y	NA	NA	Y	Y	UC	Y	Y	Y	Y	Y	Y	83.3
Moger et al. 2022 [48]	Y	Y	N	Y	Y	Y	Y	Y	Y	NA	Y	Y	Y	NA	NA	N	Y	Y	Y	Y	Y	Y	Y	Y	NA	NA	Y	Y	N	Y	Y	Y	Y	UC	Y	86.7
Sankatsing et al. 2020 [45]	Y	Y	N	Y	Y	Y	UC	Y	NA	NA	Y	Y	Y	NA	NA	N	Y	Y	Y	Y	Y	Y	Y	UC	NA	NA	Y	Y	N	Y	Y	Y	Y	Y	Y	82.8
Wang etal. 2020 [46]	Y	Y	N	Y	Y	Y	UC	Y	NA	N	Y	Y	Y	NA	NA	N	Y	Y	N	Y	Y	Y	Y	Y	NA	NA	Y	Y	Y	Y	Y	N	Y	Y	Y	80.0

N, no; NA, not available; UC, unclear; Y, yes.

### Discussion

This systematic review provides current evidence regarding the cost-effectiveness of DBT, either DBT-DM or standalone DBT, as breast cancer screening modalities for asymptomatic women at average risk. Most included studies were conducted in high-income countries, primarily in North America and Europe, and were published within the past decade. Overall, the evidence suggests that DBT-based screening strategies tend to favor improved cost-effectiveness in terms of incremental costs per QALY or LY gained, although substantial uncertainty remains across settings and modeling assumptions. The included model-based economic evaluations demonstrated heterogeneous findings, ranging from DBT being a cost-saving intervention to being cost-ineffective at conventional ICER thresholds. This variability is likely attributable to differences in healthcare system contexts, population characteristics, intervention, and methodological approaches.

### Cost-effectiveness of adjunct DBT (DBT-DM)

Most studies evaluating adjunct DBT use, particularly those conducted in North American settings [24, 25, 47], concluded that this combined screening approach was cost-effective. However, the reported ICERs varied substantially across studies. This variation may partly be explained by differences in study characteristics, particularly screening intervals, target age ranges, and healthcare costs across countries. In particular, higher healthcare expenditure in the US is likely to contribute to higher ICER estimates. An OECD analysis conducted between 2013 and 2016 showed that healthcare spending in the US was approximately twice that of other high-income countries despite similar utilization rates, largely driven by higher prices for labor, pharmaceuticals, medical goods, and administrative services [61]. However, it could be that the ICER threshold should be higher in the US, which means that the intervention could still be cost-effective, even if the ICER is higher.

Screening interval appears to be an important determinant of cost-effectiveness. The two studies evaluating biennial screening among women aged 40–69 years [13] and 40–74 years [47] reported lower ICERs compared with the study evaluating annual screening among women aged 40–79 years [25]. The higher ICER associated with annual screening may partly be attributable to a smaller absolute reduction in recall rates [25], resulting in fewer avoided false-positive cases and reduced downstream cost savings.

Evidence from US prospective cohort studies using Breast Cancer Surveillance Consortium data supports this interpretation [17, 62]. One study conducted between 1996 and 2012 found no statistically significant differences in the relative risk of less favorable invasive tumor characteristics between annual and biennial screening across age groups [62]. Another study using data from 2005 to 2018 demonstrated significantly lower 10-year cumulative probabilities of false-positive results with biennial screening compared with annual screening, including an approximate 5% absolute reduction in biopsy rates for false-positive findings [17].

The age range included in screening programs may also substantially influence ICER estimates. The study evaluating annual screening from ages 40 years to 79 years [25] reported a higher ICER than studies assessing biennial screening in women up to ages 69 years or 74 years [13, 47]. Evidence from a US population-based study using Medicare data from 2000 to 2008 showed that continuing annual mammography beyond age 75 did not significantly reduce breast cancer mortality over 8 years of follow-up [63]. This finding may reflect the greater likelihood of competing causes of mortality in older populations, implying that some screen-detected breast cancers may represent overdiagnosis [10, 63]. Importantly, overdiagnosis in older women may occur even with faster-growing cancers when life expectancy is shorter than the lead time associated with screening. Since women diagnosed with breast cancer are likely to undergo invasive treatment, overdiagnosis may attenuate the cost-effectiveness of screening in this population, particularly when treatment does not substantially improve survival outcomes.

At the same time, limiting screening to women aged 50 years and older may forgo potential benefits associated with earlier detection. A large-scale RCT conducted in the UK demonstrated that annual screening beginning at age 40 years significantly reduced breast cancer mortality at 10 years of follow-up, with a reported risk ratio of 0.75 [64]. Similarly, a recent economic evaluation conducted in Canada found that initiating biennial breast cancer screening between ages 40 years and 74 years, compared with screening from ages 50 years to 74 years, increased the number of deaths averted, LYG, and QALYs gained, while also reducing breast cancer treatment costs [65]. These findings may partly explain the relatively higher ICER observed in the study evaluating biennial screening among women aged 50–74 years [24] compared with studies initiating screening in younger populations.

### Cost-effectiveness of standalone DBT

While DBT-DM generally appeared to be cost-effective across most included studies, the evidence regarding standalone DBT remains comparatively limited, although overall findings were promising. The studies conducted in Brazil [23] and Norway [48] demonstrated that biennial DBT with s2D could represent a dominant strategy, whereby DBT screening produced greater health benefits at lower overall costs than DM alone. These favorable findings were largely attributed to lower recall rates, improved cancer detection, and reduced downstream treatment costs associated with earlier diagnosis. In the Brazilian study, the analysis focused on women aged 40–69 years with fibroglandular density and heterogeneously dense breasts [23], which represent the predominant breast density categories within this age group [66]. Despite the relatively high incremental cost of DBT based on local pricing structures, the intervention remained cost-saving because of reductions in recall rates, lower false-positive rates, and higher invasive cancer detection rates associated with DBT screening [23].

By contrast, the favorable economic outcomes observed in the Norwegian study appeared to be driven primarily by improved cancer detection rather than reductions in recall rates [48]. This may suggest greater sensitivity of DBT relative to DM within the evaluated screening setting. Using data derived from a single RCT [48, 67], the authors evaluated the general population of women aged 50–69 years. Although overall recall rates were similar between DBT and DM, cancer detection rates increased from 5.8 per 1,000 screens with DM to 7.6 per 1,000 screens with DBT [48]. Furthermore, interval cancer rates, which are often associated with less favorable prognostic characteristics, were lower with DBT than with DM [47, 48]. Earlier detection of breast cancer may therefore have contributed to both improved health outcomes and lower treatment costs, as reflected by the substantial reduction in average treatment expenditures reported in the study [48]. Supporting these findings, previous meta-analyses have consistently shown that DBT improves cancer detection rates relative to DM [15, 16, 20]. Importantly, the screening costs applied in the Norwegian model were substantially lower than those reported in several other included studies [48], which may further explain the highly favorable cost-effectiveness estimates observed.

One of the two Dutch studies reported that DBT was potentially cost-effective, with an ICER of €27,023 per LYG within the evaluated WTP threshold range of €20,000–35,000 per LYG [45]. The comparatively less robust findings may have been related to the relatively small reduction in false-positive results assumed within the model, as only modest improvements in positive predictive value were attributed to DBT screening [45]. A previous meta-analysis suggested that reductions in recall rates are more commonly observed in US screening settings, where baseline recall rates tend to be higher, whereas improvements in cancer detection rates are more frequently reported in European settings [15]. Because recall and false-positive rates associated with DM screening in the Netherlands are already relatively low, the potential reductions achievable through DBT screening may therefore be more limited [45]. In comparison, the second Dutch study [46] did not explicitly model recall rates and instead assumed constant specificity, implying no difference in false-positive rates between DBT and DM. Under these assumptions, DBT was considered cost-effective only if sensitivity reached at least 95%, a threshold exceeding that reported in most other included studies.

### Relevance to Asian screening programs

Evidence suggests that DBT improves sensitivity in dense breasts, where the performance of DM is generally more limited [20]. Because Asian women tend to have a higher prevalence of dense breasts than Western women, the gains in sensitivity and cancer detection associated with DBT may plausibly be greater in Asian populations than in the predominantly Western cohorts modeled in the identified studies. This implies that findings from Western studies may underestimate the value of DBT in Asian screening programs, particularly in terms of incremental health benefits.

Asian settings, especially LMICs, tend to face tighter resource constraints and more limited health system capacity. Several cost components are therefore likely to differ relative to those reported in the included studies. For example, lower labor costs in these settings may reduce the costs associated with both screening alternatives, whereas more limited screening infrastructure may disproportionately disadvantage the implementation of DBT. More importantly, the cost-effectiveness thresholds against which ICERs are evaluated are generally considerably lower in Asian LMICs than in the high-income settings represented in the included studies, reflecting higher health opportunity costs. Consequently, an ICER considered cost-effective in a high-income country context may still displace more productive uses of healthcare resources in an Asian LMIC, particularly given that the cost-effectiveness of DM itself remains uncertain in some of these settings [26].

### Key drivers of cost-effectiveness

Regardless of the modeling approach or healthcare setting, DSAs across the included studies consistently identified two major drivers of ICERs: reductions in recall rates and the costs associated with DBT implementation. However, the parameter ranges applied in these analyses were frequently based on assumptions that were not fully justified.

The influence of screening costs on the cost-effectiveness of DBT is intuitive. Two US studies evaluating DBT-DM identified the additional cost associated with DBT as a key determinant of cost-effectiveness outcomes, rather than parameters directly related to screening performance [24, 44]. Similarly, the Dutch study evaluating standalone DBT found that DBT-related costs substantially influenced the ICER [45]. Nonetheless, the assumptions and parameter ranges applied to DBT costs varied considerably across studies and appeared somewhat arbitrary. For example, Lee et al. [24] evaluated a broad range of additional DBT costs, from no additional cost ($0) to double the base-case cost ($139). In contrast, Lowry et al. [44] applied a narrower range, assuming DBT costs between $20 and $30 below the base-case estimate, which was sufficient to alter the study conclusion regarding cost-effectiveness. These findings underscore the importance of reducing screening costs and highlight the critical influence of DBT pricing assumptions in economic evaluations, particularly in settings where DBT pricing structures remain uncertain or have not been firmly established.

As discussed previously, reductions in recall rates may positively influence cost-effectiveness, most likely through reductions in false-positive findings and the downstream costs associated with additional diagnostic procedures and biopsies. Two studies evaluating biennial DBT-DM screening identified parameters related to recall rate reduction as the principal drivers of cost-effectiveness [13, 47]. Overall, these findings suggest that meaningful reductions in recall rates are likely necessary for DBT-based screening strategies to achieve cost-effectiveness.

### Interpretation in the context of methodological approaches

Beyond contextual differences between studies, heterogeneity in methodological approaches, particularly modeling strategies, appears to be an important determinant influencing cost-effectiveness conclusions. The included studies employed several commonly used model types, including decision trees, state-transition cohort models, microsimulations, and discrete-event simulations. Notably, most studies using patient-level simulation (PLS) models tended to report less favorable ICERs [24, 44, 45].

PLS models offer greater flexibility than cohort-based models by incorporating individual patient histories and heterogeneity in characteristics such as breast density, which may influence transitions, costs, and health outcomes [68, 69]. By more comprehensively capturing between-patient variability and disease complexity, PLS models may generate more realistic and context-sensitive cost-effectiveness estimates [68]. For example, the CISNET models used by Lowry et al. [44] incorporated detailed prognostic variables, including tumor stage and size at diagnosis, estrogen receptor and HER2 status, and age at diagnosis. It is therefore plausible that the comparatively less favorable ICERs reported in this study, relative to others, were influenced by these more nuanced assumptions, which may also have more adequately accounted for overdiagnosis and lead-time bias. Nevertheless, the use of PLS models does not necessarily lead to systematically different cost-effectiveness outcomes compared with cohort models [68]. One study evaluating adjuvant therapies for early breast cancer compared a cohort Markov model with a discrete-event simulation model and reported broadly similar ICER estimates between the 2 approaches [70].

Another important methodological consideration is the choice of time horizon, which can substantially influence cost-effectiveness results. Most included studies adopted long-term horizons [13, 23,24,25, 44,45,46,47, 49], either over a lifetime or extending for 30–40 years, whereas Moger et al. [48] applied a shorter 10-year horizon. Such a limited time horizon may be less appropriate for preventive interventions such as cancer screening, where benefits accumulate gradually and may require many years to become fully observable or to meaningfully differ between alternatives.

Despite the shorter analytic horizon, DBT remained a dominant strategy in the study by Moger et al. [48], supporting the robustness of the findings. Moreover, extending the time horizon to 20 years further improved the ICER in favor of DBT. Similarly, Chung et al. [13] found that shorter time horizons of 10 years and 20 years increased ICERs to $14,645 and $8,132 per QALY gained, respectively. Consistent with these findings, a US systematic review reported that longer time horizons generally produced more favorable ICER estimates for screening interventions [71]. Taken together, these findings highlight the importance of adopting sufficiently long time horizons in economic evaluations to capture all meaningful differences in costs and health outcomes between screening alternatives [69]. For future studies, lifetime horizons should ideally be considered the standard approach for breast cancer screening evaluations in order to fully capture the long-term benefits associated with earlier cancer detection.

### Reporting quality

A common limitation across the identified studies was the insufficient justification for the parameter ranges and assumptions applied in DSAs, with several analyses relying on arbitrary values. Ideally, parameter ranges should be derived from empirical evidence, such as 95% confidence interval or probability distributions informed by underlying data [72]. The lack of empirical justification limits the robustness of conclusions regarding the identified key drivers of cost-effectiveness, particularly DBT costs and recall rates. Because the influence of a parameter on the ICER depends partly on the magnitude over which it is varied, poorly justified ranges may distort which parameters appear most influential within sensitivity analyses. Consequently, these findings should be interpreted with caution. Additionally, most identified CUA studies provided limited information regarding the valuation methods and the characteristics of the populations from which utility estimates were derived, although a small number of studies explicitly specified the use of EQ-5D for utility measurement [23, 24].

### Strengths and limitations

The present review has several strengths. First, it specifically focused on the cost-effectiveness of DBT, whether used as an adjunct to DM or as a standalone screening modality, compared with DM alone. In contrast, previous reviews have generally examined broader breast cancer screening approaches. As a result, this review is directly relevant to ongoing international policy discussions concerning the health opportunity costs associated with implementing DBT in routine population-based screening programs. Second, the review provided a narrative synthesis of cost-effectiveness findings across studies in relation to both study characteristics, such as target age groups and screening intervals, and methodological approaches. This approach may help inform clinical practice, policy decision-making, and future research priorities. Finally, the review employed a comprehensive search strategy using detailed search terms across 7 databases and followed established best-practice guidance for systematic review conduct and reporting wherever feasible.

Several limitations should also be acknowledged. First, the review was conducted by a single author (SC), including study selection, data extraction, quality assessment, and data synthesis. This may have increased the risk of errors or subjective bias, particularly given that these processes often involve interpretive judgment. However, these risks were likely mitigated through regular discussions with the project supervisor (NA) and by systematically adhering to established methodological guidance [29]. Second, the review was limited to peer-reviewed literature and excluded gray literature, thereby introducing the potential for publication bias. Nevertheless, the review identified several relevant economic evaluations of DBT that were not captured in previous reviews. The exclusion of gray literature was a pragmatic decision intended to maintain a manageable review scope within the project timeline while preserving transparent and reproducible eligibility criteria. During study selection, a Canadian HTA report evaluating supplemental screening adjunctive to mammography for breast cancer screening was identified [42], but excluded because it did not meet the predefined eligibility criteria. Finally, most included studies were conducted in North America [24, 25, 44, 47] and Europe [45, 46, 48, 49], and only one study focused exclusively on women with dense breasts [24]. These factors limit the generalizability of the findings to other healthcare settings, particularly LMICs with more constrained resources, as well as to diverse ethnic populations and clinically relevant subgroups such as women with dense breast tissue.

## Conclusions

Findings from this systematic review suggest that breast cancer screening using DBT in the general population may provide good value for healthcare systems, although cost-effectiveness appears to depend substantially on how screening is implemented and which populations are targeted. Although DBT has been increasingly adopted worldwide, the available economic evidence remains concentrated in high-income countries, and economic evaluations focusing specifically on women with dense breasts remain limited. Further robust economic evaluations across more diverse populations and healthcare system contexts, particularly within Asian settings, are needed to support broader policy and implementation decisions.

## Appendix 1. Search strategies

### 1. Embase (Ovid)

Segment searched: 1974 to June 18, 2025

Date searched: June 19, 2025

Number of records found: 363

Search strategies and search hits:

**Table:**

1. digital breast tomosynthesis/	2537
2. (“3-D mammogra$” or “3D mammogra$” or “three dimensional mammogra$” or “3-dimensional mammogra$”).ti,ab.	146
3. (tomosynthesis adj6 (breast$ or mammar$)).ti,ab.	2413
4. (“3Dimensions” or “Selenia Dimensions” or “Senographe Pristina” or “VolumeRAD” or “Wide-Angle Tomosynthesis” or “Helianthus” or “ASPIRE Cristalle” or “Amulet Innovality” or “Planmed Clarity” or “Giotto Class”).ti,ab.	2853
5. Health Economics/	37479
6. exp Economic Evaluation/	395752
7. exp Health Care Cost/	373813
8. pharmacoeconomics	22712
9. 5 or 6 or 7 or 8	702379
10. (econom$ or cost or costs or costly or costing or price or prices or pricing or pharmacoeconomic$).ti,ab.	1628716
11. (expenditure$ not energy).ti,ab.	56798
12 (value adj2 money).ti,ab.	3324
13 budget$.ti,ab.	53134
14 10 or 11 or 12 or 13	1678856
15 9 or 14	1948472
16 letter.pt.	` 1367862
17 editorial.pt.	840046
18 note.pt.	1011807
19 16 or 17 or 18	3219715
20 15 not 19	1823610
21 (metabolic adj cost).ti,ab.	2102
22 ((energy or oxygen) adj cost).ti,ab.	5543
23 ((energy or oxygen) adj expenditure).ti,ab.	42102
24 21 or 22 or 23	48417
25 20 not 24	1813783
26 animal/	1714835
27 exp animal experiment/	3376239
28 nonhuman/	8196933
29 (rat or rats or mouse or mice or hamster or hamsters or cat or cats or bovine or sheep).ti,ab,sh.or animal or animals or dog or dogs	6980972
30 26 or 27 or 28 or 29	11257887
31 exp human/	28645138
32 human experiment/	719122
33 31 or 32	28648462
34 30 not (30 and 33)	7810731
35 25 not 34	1629519
36 1 or 2 or 3 or 4	6081
37 35 and 36	363

### 2. MEDLINE (Ovid)

Segment searched: 1946 to June 18, 2025

Date searched: June 19, 2025

Number of records found: 283

Search strategies and search hits:

**Table:**

1 (“3-D mammogra$” or “3D mammogra$” or “three dimensional mammogra$” or “3-dimensional mammogra$”).ti,ab.	74
2 (tomosynthesis adj6 (breast$ or mammar$)).ti,ab.	1861
3 (“3Dimensions” or “Selenia Dimensions” or “Senographe Pristina” or “VolumeRAD” or “Wide-Angle Tomosynthesis” or “Helianthus” or “ASPIRE Cristalle” or “Amulet Innovality” or “Planmed Clarity” or “Giotto Class”).ti,ab.	3047
4 1 or 2 or 3	4891
5 Economics/	27549
6 exp “costs and cost analysis“/	279639
7 Economics, Dental/	1922
8 exp economics, hospital/	26225
9 Economics, Medical/	9306
10 Economics, Nursing/	4013
11 Economics, Pharmaceutical/	3164
12 (economic$ or cost or costs or costly or costing or price or prices or pricing or pharmacoeconomic$).ti,ab.	1220002
13 (expenditure$ not energy).ti,ab.	41338
14 value for money.ti,ab.	2399
15 budget$.ti,ab.	39892
16 or/5–15	1392581
17 ((energy or oxygen) adj cost).ti,ab.	5151
18 (metabolic adj cost).ti,ab.	1893
19 ((energy or oxygen) adj expenditure).ti,ab.	31483
20 or/17–19	37408
21 16 not 20	1383880
22 exp animals/not humans/	5349190
23 21 not 22	1296959
24 4 and 23	283

### 3 EB Health - KSR Evidence via Ovid

Segment searched: 2015 to 2025 Week 24

Date searched: June 19, 2025

Number of records found: 54

Search strategies and search hits:

**Table:**

1 (“3-D mammogra$” or “3D mammogra$” or “three dimensional mammogra$” or “3-dimensional mammogra$”).ti,ab.	3
2 (tomosynthesis adj6 (breast$ or mammar$)).ti,ab.	53
3 (“3Dimensions” or “Selenia Dimensions” or “Senographe Pristina” or “VolumeRAD” or “Wide-Angle Tomosynthesis” or “Helianthus” or “ASPIRE Cristalle” or “Amulet Innovality” or “Planmed Clarity” or “Giotto Class”).ti,ab.	1
4 1 or 2 or 3	54

### 4. Cochrane Library via Wiley

Segment searched: inception to present

Date searched: June 19, 2025

Number of records found: 101

Search strategies and search hits:

**Table:**

#1 ((3D NEXT mammogra*) or (3 NEXT D NEXT mammogra*) or (three NEXT dimensional NEXT mammogra*) or (3 NEXT D NEXT mammogra*)):ti,ab	3
#2 ((tomosynthesis NEAR/6 breast*) or (tomosynthesis NEAR/6 mammar*)):ti,ab	76
#3 (3Dimensions or “Selenia Dimensions” or “Senographe Pristina” or “VolumeRAD” or “Wide-Angle Tomosynthesis” or Helianthus or “ASPIRE Cristalle” or “Amulet Innovality” or “Planmed Clarity” or “Giotto Class”):ti,ab	22
#4 #1 or #2 or #3	101

### 5. NHS EED

Segment searched: inception to the end of March 2015 (ceased)

Date searched: June 19, 2025

Number of records found: 1

Search strategies and search hits:

**Table:**

(“3-D mammogram” or “3-D mammograms” or “3-D mammography” or “3-D mammographies” or “3D mammogram” or “3D mammograms” or “3D mammography” or “3D mammographies” or “three dimensional mammogram” or “three dimensional mammograms” or “three dimensional mammography” or “three dimensional mammographies” or “three-dimensional mammogram” or “three-dimensional mammograms” or “three-dimensional mammography” or “three-dimensional mammographies”) OR (tomosynthesis)	1

### 6. CEA registry

Segment searched: inception to present

Date searched: June 19, 2025

Number of records found: 6

Search strategies and search hits:

**Table:**

“3-D mammogram” OR “3-D mammograms” OR “3-D mammography” OR “3-D mammographies” OR “3D mammogram” OR “3D mammograms” OR “3D mammography” OR “3D mammographies” OR “three dimensional mammogram” OR “three dimensional mammograms” OR “three dimensional mammography” OR “three dimensional mammographies” OR “three-dimensional mammogram” OR “three-dimensional mammograms” OR “three-dimensional mammography” OR “three-dimensional mammographies” OR tomosynthesis	6

### 7. RePEc IDEAS

Segment searched: inception to present

Date searched: June 19, 2025

Number of records found: 21

Search strategies and search hits:

**Table:**

“3-D mammogram” | “3-D mammograms” | “3-D mammography” | “3-D mammographies” | “3D mammogram” | “3D mammograms” | “3D mammography” | “3D mammographies” | “three dimensional mammogram” | “three dimensional mammograms” | “three dimensional mammography” | “three dimensional mammographies” | “three-dimensional mammogram” | “three-dimensional mammograms” | “three-dimensional mammography” | “three-dimensional mammographies” | tomosynthesis	21

## Appendix 2. The BMJ checklist

The 35-point checklist is as follows [1]:

1) The research question is stated
2) The economic importance of the research question is stated
3) The viewpoint(s) of the analysis are clearly stated and justified
4) The rationale for choosing the alternative programmes or interventions compared is stated
5) The alternatives being compared are clearly described
6) The form of economic evaluation used is stated
7) The choice of form of economic evaluation is justified in relation to the questions addressed
8) The source(s) of effectiveness estimates used are stated
9) Details of the design and results of effectiveness study are given (if based on a single study)
10) Details of the method of synthesis or meta-analysis of estimates are given (if based on an overview of a number of effectiveness studies)
11) The primary outcome measure(s) for the economic evaluation are clearly stated
12) Methods to value health states and other benefits are stated
13) Details of the subjects from whom valuations were obtained are given
14) Productivity changes (if included) are reported separately
15) The relevance of productivity changes to the study question is discussed
16) Quantities of resources are reported separately from their unit costs
17) Methods for the estimation of quantities and unit costs are described
18) Currency and price data are recorded
19) Details of currency of price adjustments for inflation or currency conversion are given
20) Details of any model used are given
21) The choice of model used and the key parameters on which it is based are justified
22) Time horizon of costs and benefits is stated
23) The discount rate(s) is stated
24) The choice of rate(s) is justified
25) An explanation is given if costs or benefits are not discounted
26) Details of statistical tests and confidence intervals are given for stochastic data
27) The approach to sensitivity analysis is given
28) The choice of variables for sensitivity analysis is justified
29) The ranges over which the variables are varied are stated
30) Relevant alternatives are compared
31) Incremental analysis is reported
32) Major outcomes are presented in a disaggregated as well as aggregated form
33) The answer to the study question is given
34) Conclusions follow from the data reported
35) Conclusions are accompanied by the appropriate caveats
